# Precise segmentation of densely interweaving neuron clusters using G-Cut

**DOI:** 10.1038/s41467-019-09515-0

**Published:** 2019-04-04

**Authors:** Rui Li, Muye Zhu, Junning Li, Michael S. Bienkowski, Nicholas N. Foster, Hanpeng Xu, Tyler Ard, Ian Bowman, Changle Zhou, Matthew B. Veldman, X. William Yang, Houri Hintiryan, Junsong Zhang, Hong-Wei Dong

**Affiliations:** 1 Fujian Key Laboratory of Brain-Inspired Computing Technique and Applications, Department of Cognitive Science, School of Informatics, Xiamen University, Xiamen, 361005 China; 2 Center for Integrative Connectomics, USC Stevens Neuroimaging and Informatics Institute, Keck School of Medicine of USC, University of Southern California, Los Angeles, CA 90095 USA; 3 National Engineering Research Center for E-Learning, Central China Normal University, 430079 Wuhan, China; 4 Present Address: Intuitive Surgical Inc., 1020 Kifer Road, Sunnyvale, CA 94086 USA; 5 Laboratory of Neuroimaging, USC Stevens Neuroimaging and Informatics Institute, Keck School of Medicine of USC, University of Southern California, Los Angeles, CA 90095 USA; 6 Center for Neurobehavioral Genetics, Jane and Terry Semel Institute for Neuroscience and Human Behavior, University of California at Los Angeles, Los Angeles, CA 90095 USA; 7 Department of Psychiatry and Biobehavioral Sciences, David Geffen School of Medicine at UCLA, Los Angeles, CA 90095 USA; 8 Zilkha Neurogenetic Institute, University of Southern California, Los Angeles, CA 90095 USA; 9 Department of Neurology, Keck School of Medicine of USC, University of Southern California, Los Angeles, CA 90095 USA

## Abstract

Characterizing the precise three-dimensional morphology and anatomical context of neurons is crucial for neuronal cell type classification and circuitry mapping. Recent advances in tissue clearing techniques and microscopy make it possible to obtain image stacks of intact, interweaving neuron clusters in brain tissues. As most current 3D neuronal morphology reconstruction methods are only applicable to single neurons, it remains challenging to reconstruct these clusters digitally. To advance the state of the art beyond these challenges, we propose a fast and robust method named G-Cut that is able to automatically segment individual neurons from an interweaving neuron cluster. Across various densely interconnected neuron clusters, G-Cut achieves significantly higher accuracies than other state-of-the-art algorithms. G-Cut is intended as a robust component in a high throughput informatics pipeline for large-scale brain mapping projects.

## Abstract (web summary)

Most neuronal reconstruction software can automatically trace single neuronal morphologies but tracing multiple, densely interwoven neurons is much more challenging. Here the authors develop G-Cut, a computational approach for accurate segmentation of densely interconnected neuron clusters.

## Introduction

Enumerating and characterizing the diversity of neuronal cell types has been posed as one of the major challenges of the BRAIN Initiative, with the vision that such classification is an important step toward dissecting their functional contributions in health and disease (http://braininitiative.nih.gov/). Although no consensus has been reached for a satisfactory definition of neuronal cell types^1,2^, it is generally agreed that neuronal morphology, together with anatomic location, structural connectivity and gene expression profiles are significant discriminating elements^3,4^. The reconstruction of individual neuronal dendrites and axons enables quantitative analysis of morphological features of a given neuron type to provide insight into its functional role. The numbers and lengths of dendritic arbors of a given neuron serve as an indication of its receptive field for receiving information, while axonal trajectory and projection fields determine neuronal outputs that regulate neural activities of its targeted neurons^5–7^. Furthermore, morphological abnormalities of neurons are usually correlated with pathological changes in numerous neurological disorders such as Alzheimer’s disease, Huntington’s disease, and autism^8^. Therefore, characterizing fine detailed neuronal morphology is crucial for classifying neuronal cell types and understanding their functional attributes.

With recent advancements in tissue clearing and microscopic technologies, new challenges and opportunities arise in reconstructing detailed neuronal morphology. High-resolution image stacks from large tissue blocks of intact brain tissue can now be routinely acquired, providing valuable information about three-dimensional neuronal morphology in intact neural circuitry. In such contiguously imaged tissues, researchers frequently encounter densely labeled (either genetically or with viral tracers), interweaving neuron clusters with intermingled dendritic arbors and axonal branches of multiple neurons. Existing automatic reconstruction software are mostly designed to automatically and elegantly trace single neuronal morphologies, but it still remains challenging to apply these reconstruction software to correctly trace multiple neurons that are densely interwoven. Therefore, computational approaches are required to obtain accurate reconstructions from image stacks where signal is contributed by multiple neurons with substantial spatial intermingling^9^.

In this study, we report a computational approach called G-Cut to address the challenge of reconstructing densely interwoven neurons. G-Cut is able to automatically segment individual neurons robustly from neuron clusters based on combinatorial information of morphological features and connections among intermingled neurites. The estimation of relatedness between individual neurites and somas is based on the morphological information statistically derived from existing large neuronal morphological datasets (i.e., NeuroMorpho.Org). Using both synthetic datasets and real image stacks from tissue blocks, we demonstrate that, in comparison with other state-of-the-art algorithms, G-Cut achieves higher accuracy in individual neuron segmentation across neuron clusters of various labeling densities and morphological patterns. In short, G-Cut is a robust and powerful informatics tool with broad applications in morphology reconstructions of large numbers of neurons and consequently accelerates the process of cataloging neuronal cell types of the brain.

## Results

### General workflow of G-Cut

**Fig. 1:**
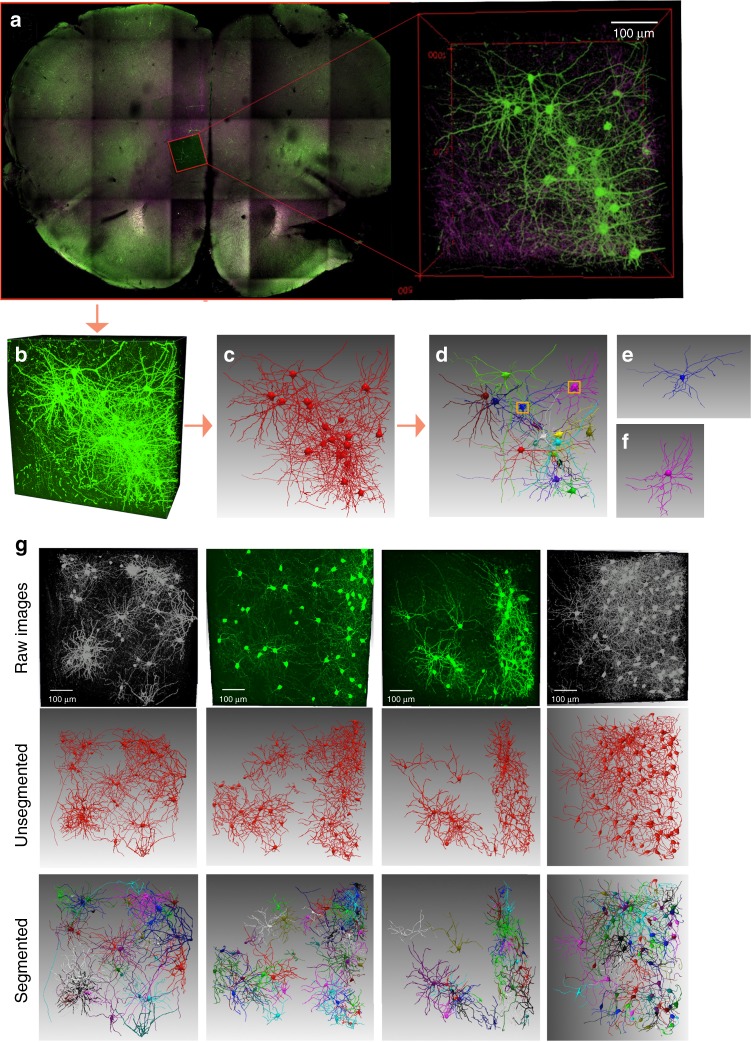
A workflow for automated reconstruction of individual neurons from a neuron cluster. **a** An image stack with a densely labeled neuron cluster is obtained with appropriate tissue preparation and imaging techniques (e.g., confocal microscopy, multiphoton microscopy on clarified tissue, etc.). **b** An existing tracing method (e.g., NeuronStudio software) is applied to obtain an unsegmented reconstruction of the neural cluster as shown in **c**. G-Cut is applied to the unsegmented reconstruction. Resulting individual neurons are shown with distinct colors as shown in **d**. **e** and **f** show enlarged view of two reconstructed single neurons corresponding to the orange boxes in **d**. **g** shows segmentation result of G-Cut on four reconstructed neuron clusters. Initial unsegmented neuron clusters were reconstructed from experimental image stacks with APP2 algorithm in Vaa3D software. The result shows that G-Cut can be applied on clusters with numerous neurons

**Fig. 2:**
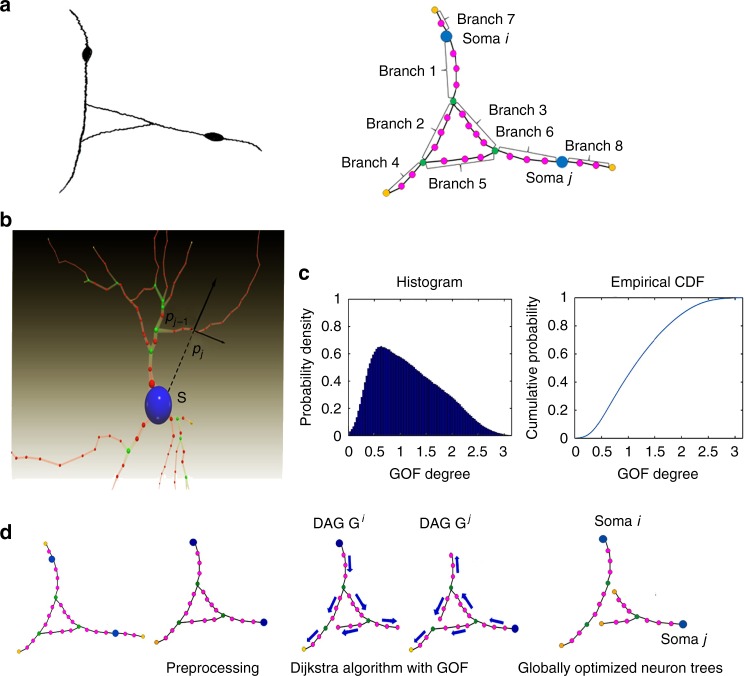
G-Cut performs automatic segmentation based on biological features and graph theory. **a** The original graph representation of a neuron cluster consists of branches and somas. Each branch starts at one topological node, and ends at another topological node (soma nodes shown as blue, branch nodes shown as green, leaf nodes shown as yellow, path nodes shown as magenta, and edges between nodes shown as solid line). **b** The Growth Orientation Feature (GOF) quantifies the orientation of a branch with respect to a soma. Blue node: soma. Green node: branch node. Yellow node: leaf node. Red node: path node. **c** Left-hand panel shows an example histogram of the GOF for more than 70,000 experimentally reconstructed neuronal morphologies from the NeuroMorpho.Org database. Right-hand panel shows cumulative distribution function of the GOF derived from its histogram. **d** We identify bridging branches between somas and establish their topological relations by constructing a directed acyclic graph (DAG) rooted at a given soma using the Dijkstra algorithm. The branches which are reachable from only one soma are excluded with a Breadth-first search algorithm in preprocessing to reduce problem size. The direction of each branch and its path from the soma are established in this step (direction of each branch shown as blue arrow). After that, the linear programming algorithm is used to find a globally optimal segmentation for the neuron cluster

G-Cut is designed as a component of an informatics pipeline to reconstruct populations of neurons with intermingling neurites in 3D images (Fig. 1). In G-Cut, a connected graph representation of a neuron cluster (consisting of interwoven neurons) is given as input to the algorithm. The graph contains neuronal cell bodies and branches whose correct cell body assignment has not yet been determined (Fig. 2a). In practice, any known single neuron reconstruction algorithm, for example, Vaa3D by Peng et al.^10,11^, can be used to obtain the input graph.

Segmentation is achieved by finding a set of globally optimal branch assignments over all possible topological connectivity configurations of the graph. In this optimization problem, a metric is needed to evaluate the fitness of branch assignments. When viewing reconstruction results from manual tracing, we observe that neurites often follow a locally smooth path, extending along the current orientation (inertia) and away from the soma (tropism)^12^. To capture these properties, a Growth Orientation Feature (GOF) metric is used to described the branch orientation deviation in relation to a given soma (Methods and Fig. 2b). The expected distribution of this feature is empirically derived using more than 70,000 neurons in a standard public database available from NeuroMorpho.Org^4^ (Fig. 2c).

**Fig. 3:**
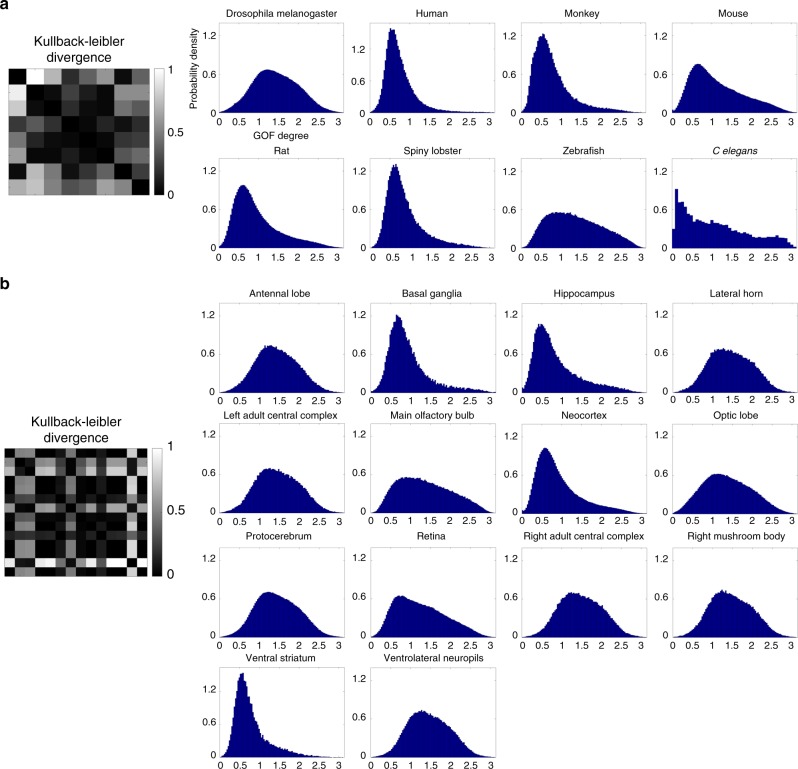
GOF distributions obtained from public datasets available on the NeuroMorpho.Org website. Neurons are cataloged by species or by brain regions. **a** GOF distribution of each species contains more than one thousand neurons (excluding *C. elegans* which only has 424 neurons in total). **b** GOF distribution of each brain region contains more than one thousand neurons. Kullback-Leibler divergence was performed on different species and different brain regions, respectively

Considering that different cell types display rich varieties of morphologies in different species (e.g., *C elegans*, mouse, rat, monkey, and human) or in different brain structures (e.g., retina, neocortex and main olfactory bulb), we computed the distributions of GOF, respectively, in a total of 8 different species and 14 different brain structures (Fig. 3). These GOF distributions allow users to segment neuron clusters of different cell types according to species or structural characteristics. To increase its applicability, G-Cut also allows users to obtain the distribution of the GOF from customized neuronal morphology datasets provided for specific research purposes, allowing further computations to be performed on the most appropriate cell types (Supplementary Fig. 1). Unless otherwise stated, G-Cut employs a single parameter that specifies which of the species or brain region specific GOF distributions to be used (or that a custom GOF distribution should be calculated). Subsequently, this computed GOF distribution is used to quantify the fitness of each branch-soma pair to enable data driven determination of branch-soma assignments.

G-Cut calculates the complete set of segmentation configurations in a neuron cluster in order to calculate the global optimum. Topological relations between branches under different segmentation configurations are derived with well-established graph algorithms and translated to a series of algebraic constraints (Methods and Fig. 2d). An optimization process that maximizes the global GOF based fitness of all soma branch pairs can then partition the graph into individual neurons. We use a linear programming approach^13^ to solve the optimization problem because: (1) the optimized function is linear in nature (Methods); (2) a global optimum is guaranteed to be found (there is no conflicting constraint in our system); and (3) many fast and reliable solvers are available. Because the distribution of GOF, which the fitness metric is based on, is derived from real world biological data, our method achieves biologically valid segmentation of individual neurons.

### Evaluation of G-Cut accuracy with simulated neuron clusters

To our knowledge, only two other algorithms have been proposed to address the challenge of multi-neuron reconstruction. One is TREES toolbox, which uses a growth competition algorithm to reconstruct multiple neurons with minimum wiring cost^14^. Another method, NeuroGPS-Tree locates neuronal cell bodies^15^ and uses statistical distribution of several biological features to determine break points between bridged neurons^16^. Although these two algorithms can reconstruct individual neurons from a neuron cluster, they have several limitations for reconstructing densely interweaving neurons. Output of TREES toolbox is highly parameterization dependent. Both TREES toolbox and NeuroGPS-Tree rely on local morphological features and are therefore unable to determine the most probable partition in the entire connected graph.

**Fig. 4:**
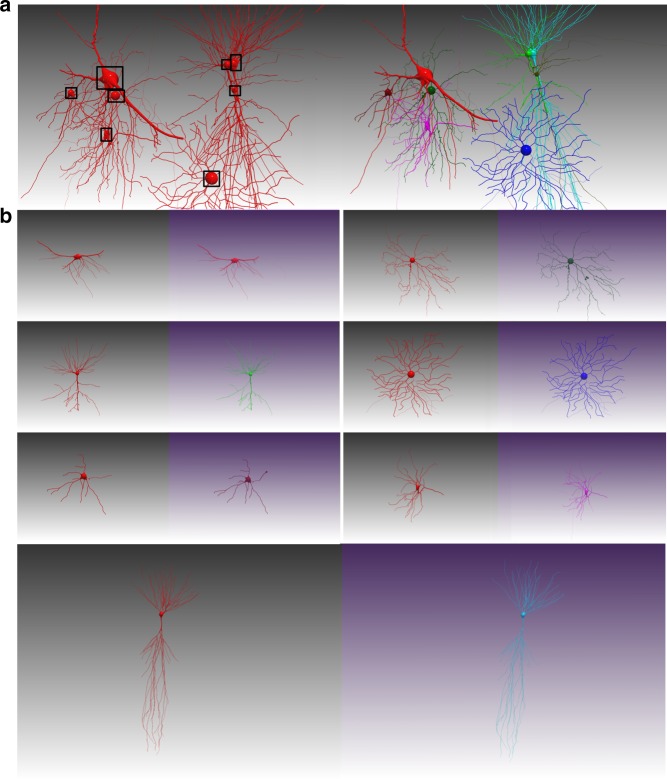
Segmentation accuracy of G-Cut on a simulated neuron cluster. **a** A simulated neuron cluster (left panel) and segmentation result (right panel). Each segmented neuron is given a unique color for easier visualization. **b** Comparison between each original neuron (left) and segmented neuron (right). The neurons are rotated for more effective visual presentation. Rendering is done with neuTube software

To systematically evaluate the accuracy of G-Cut and compare it with NeuroGPS-Tree and TREES toolbox, we investigated the accuracy of all three methods with respect to two characteristics of a neuron cluster: cluster scale and degree of inter-neuron entanglement. Cluster scale refers to the number of neurons in a cluster. Cluster degree of entanglement is represented by the number of spurious links bridging proximal neurons. Both characteristics change with experimental protocols (e.g., volume of the tissue, density of neuronal labeling, etc.) and influence the difficulty of the segmentation problem. We created two large synthetic datasets of simulated neuron clusters of varying scale and degree of entanglement, respectively, using reconstructed single neurons hosted on NeuroMorpho.Org. Briefly, single neurons were randomly placed in space, where any pair of branches in close proximity was subsequently connected, bridging neurons into a cluster (Fig. 4, Supplementary Note 1 and Supplementary Fig. 2). This allowed the rapid evaluation of the outcomes of each algorithm against an established ground truth. We measured the accuracy of neuron segmentation results with the Miss-Extra-Scores (MES)^17^, which quantifies topological resemblance between segmented and ground truth neurons.

**Fig. 5:**
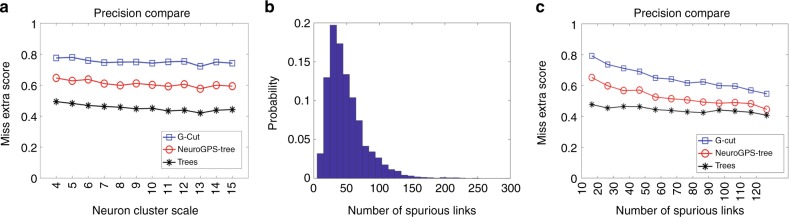
Evaluating the segmentation accuracy of G-Cut on simulated neuron clusters. **a** Segmentation accuracy of G-Cut compared to NeuroGPS-tree and TREES toolbox on different scales of simulated neuron clusters (sample size: 100 neuron clusters for each scale). Mann–Whitney *U* tests with Benjamini-Hochberg correction show that G-Cut has consistently higher accuracy (*p* < 0.01) across all scales of clusters. **b** The probability distribution of degree of entanglement in randomly generated neuron clusters with a fixed cluster scale of six. **c** Segmentation accuracy of G-Cut compared to NeuroGPS-Tree and TREES toolbox on simulated neuron clusters with different degrees of entanglement (sample size: 100 neuron clusters for each degree of entanglement). The MES result of G-Cut is consistently higher than both NeuroGPS-TREE and TREES toolbox at all degrees of entanglement (*p* < 0.01, Mann–Whitney *U* test with Benjamini-Hochberg correction). Source data are provided as a Source Data file

To understand how cluster scale affects segmentation accuracy, we formed clusters with increasing number of individual neurons, with upper bounds on cluster entanglement with the empirical distribution of spurious link numbers between randomly placed neuron pairs (Supplementary Note 1). MES following G-Cut segmentation remained high across various cluster scales, and we did not find obvious deterioration in MES results as the cluster scale increased (Fig. 5a and Supplementary Fig. 3a). Details of the statistical analysis are given in Supplementary Fig. 4a. Further, in comparison with NeuroGPS-Tree and TREES toolbox, G-Cut had higher MES scores across all scales of simulated neuron clusters (Mann–Whitney *U* tests with Benjamini-Hochberg correction, *p* < 0.01, Fig. 5a).

To understand how cluster degree of entanglement affects segmentation accuracy, we formed clusters with a constant number of individual neurons, while leaving the spurious link number in clusters unbound. Since the distribution of spurious links is a non-uniform value where events with very high link numbers occur at low frequency (Fig. 5b), we generated a large number of clusters at fixed scale and performed stratified sampling at varying degrees of entanglement (Supplementary Note 1). As the cluster degree of entanglement increases, MES results of G-Cut, NeuroGPS-tree and TREES toolbox were all seen to decrease (Fig. 5c, Supplementary Figs. 3b and 4b). However, the MES of G-Cut was still higher than that of the two other algorithms across all degrees of entanglement (Mann–Whitney *U* tests with Benjamini-Hochberg correction, *p* < 0.01, Fig. 5c).

With these two datasets, we demonstrated that G-Cut has high segmentation accuracy and outperforms both NeuroGPS-Tree and TREES toolbox in challenging neuron clusters with large scale or high degree of entanglement.

### Validation of G-Cut performance on densely connected neurons

**Fig. 6:**
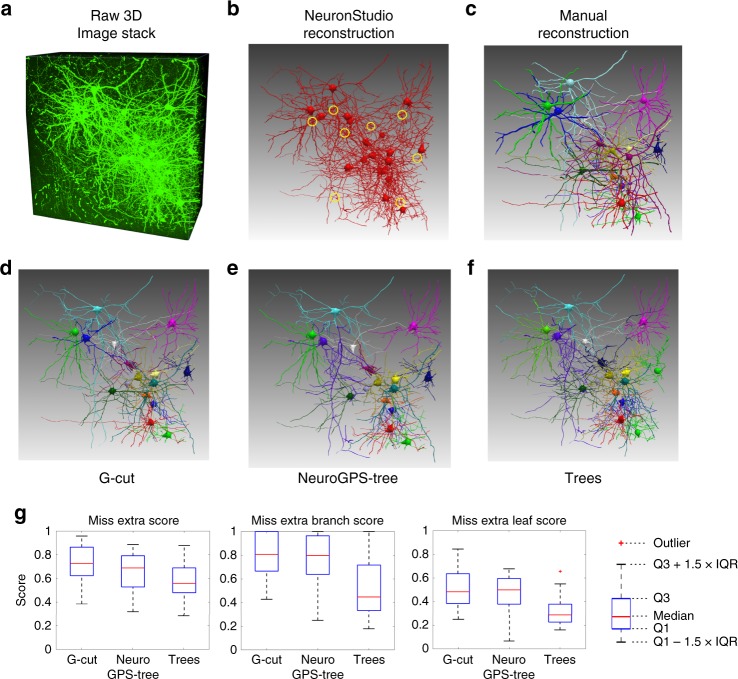
Performance of G-Cut, NeuroGPS-tree and TREES toolbox on densely connected neurons. **a** The raw image stack. Data size: 1024 × 1024 × 500 voxels. **b** A neuron cluster was automatically reconstructed from the image stack by NeuronStudio software. The neuron cluster contained 108 spurious links in total. Some representative spurious links were marked in yellow circles. **c** Sixteen neurons were manually reconstructed from the raw image stack with neuTube software and used as ground truth. **d**–**f** The neuron cluster in **b** was segmented into individual neurons by G-Cut, NeuroGPS-tree and TREES toolbox, respectively. Identical post-processing was used on segmentation results from all three algorithms (see Supplementary Fig. 7 and Supplementary Note 2). **g** Miss-Extra-Scores of the sixteen neurons reconstructed by G-Cut, NeuroGPS-Tree and TREES toolbox. The MES was obtained by comparing an automatic reconstruction result with the manual reconstruction result of each neuron. The red line represents median MES. Source data are provided as a Source Data file

To validate our results from synthetic datasets on real image stacks, we evaluated the performance of G-Cut, NeuroGPS-Tree and TREES toolbox on an image volume containing densely interconnected neurons (Fig. 6a). A neuron cluster graph was reconstructed using NeuronStudio software^18^ (Fig. 6b). The reconstructed neuron cluster was segmented by the three methods: G-Cut, NeuroGPS-Tree and TREES toolbox. Additionally, manual tracing of individual neurons was performed with neuTube software to establish ground truth^19^ (Fig. 6c). The MES comparison of segmented and ground truth neurons showed the result of G-Cut to be close to ground truth and more accurate than NeuroGPS-Tree and TREES toolbox (Fig. 6d–g). We tested all three algorithms on an additional densely labeled image stack, with results demonstrating high accuracy by G-Cut segmentation (Supplementary Fig. 5). These results are consistent with findings from synthetic neuron clusters with high degree of entanglement, and demonstrate the reliability of G-Cut as a component in an informatics reconstruction pipeline applied toward densely labeled neuronal morphologies in image stacks.

### Validation of G-Cut performance on large number of neurons

**Fig. 7:**
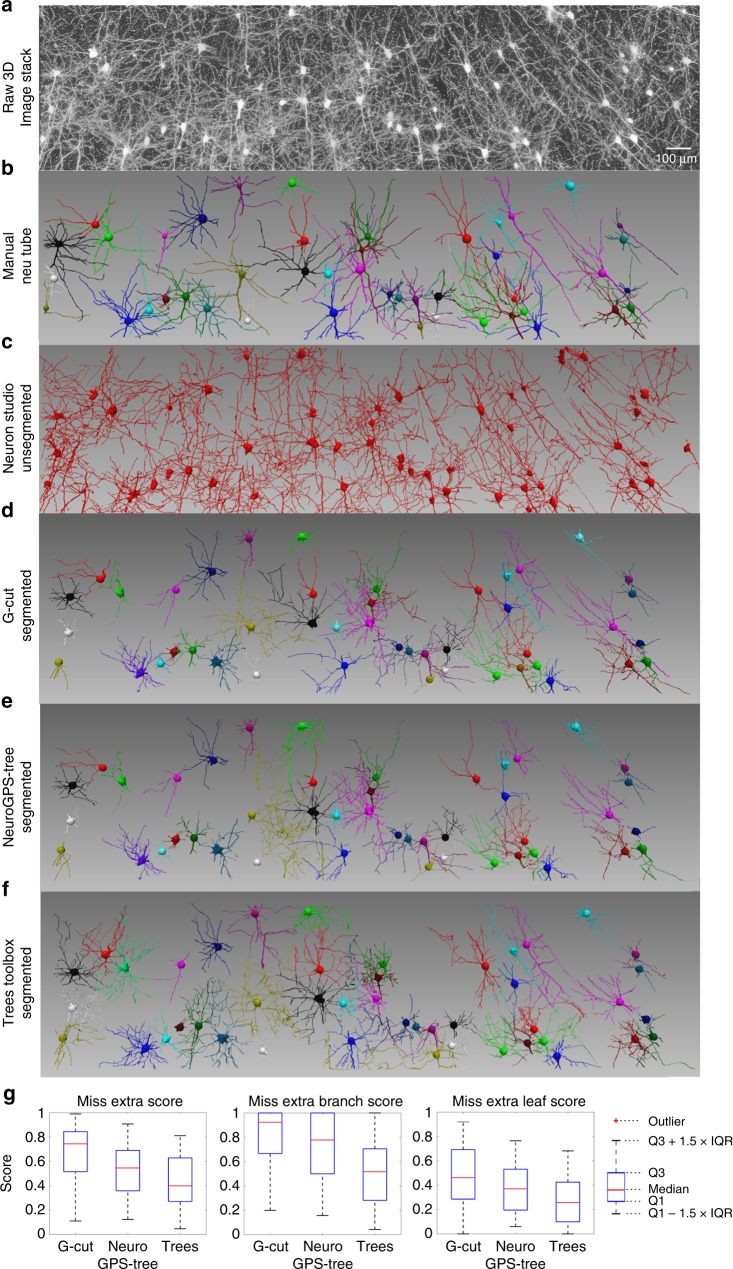
Validation of G-Cut, NeuroGPS-tree and TREES toolbox on a large number of neurons. **a** The raw Golgi-cox staining image stack. Data size: 8192 × 2048 × 46 voxels. **b** Forty-five neurons were manually reconstructed using neuTube software and used as ground truth. **c** Neuron clusters were reconstructed from the image stack by automatic tracing methods in NeuronStudio software. The neuron clusters contained 169 spurious links in total. **d**–**f** The neuron clusters in **c** were segmented by G-Cut, NeuroGPS-tree and TREES tool box into 45 individual neurons, respectively. Identical post-processing was applied to results from all three algorithms to fix tracing errors and prune redundant branches (see Supplementary Fig. 7 and Supplementary Note 2). **g** Miss-Extra-Scores of the forty-five neurons segmented by G-Cut, NeuroGPS-Tree, and TREES toolbox. Source data are provided as a Source Data file

We further tested the performance of G-Cut and compared it with NeuroGPS-Tree and TREES toolbox on an image stack containing large numbers of intermingling neurons. The image stack was obtained by classic Golgi-cox staining (Fig. 7a). In addition to the numerous neuronal cell bodies and their extended neurites, the image stack also contained many neurites originating from locations outside of the imaged volume, both characteristics causing tracing errors when existing automatic tracing methods were applied (Supplementary Fig. 6). Therefore, firstly, we manually reconstructed forty-five neurons as ground truth using neuTube software (Fig. 7b). Then, we used automatic tracing methods in NeuronStudio software to reconstruct neuron clusters from the image stack (Fig. 7c). Subsequently, these neuron clusters were segmented using G-Cut, NeuroGPS-tree and TREES toolbox into 45 individual neurons, respectively (Fig. 7d–f). Our results showed that the MES of G-Cut segmentation result remained robust even when the reconstructed neuron cluster contained tracing errors, and was more accurate than the segmentation results of NeuroGPS-Tree and TREES toolbox (Fig. 7d–g).

However, we emphasize that we conducted our image stack segmentation experiments on the best tracing results attainable by state-of-the-art algorithms. Topological tracing errors in upstream automatic tracing methods decrease the quality of input received by G-Cut and can in turn negatively impact its output (Supplementary Figs. 6–8 and Supplementary Note 2). Although G-Cut can correct some of the topological errors at present, some tracing inaccuracies remain and are visible in the output of G-Cut. Therefore, methods that detect and rectify topological errors in automatically traced neuron clusters with high success rates are needed to optimize the performance of G-Cut. Development of these error correction methods is planned for our future technology work.

## Discussion

Characterizing precise three-dimensional neuronal morphology and anatomical context of neurons is crucial for neuronal cell type classification and circuitry mapping. Achieving this requires technological advancements in three areas: (1) More efficient and robust labeling methods, for example, Brainbow^20,21^ and recently developed sparse labeling technologies^22^. These technologies fully reveal the detailed neuronal morphologies of single neurons that are distinguishable from a tangle of numerous neuronal processes of numerous other neurons; (2) Microscopic imaging technologies that enable imaging high-resolution 3D neuronal morphologies in tissue blocks or even whole brains; (3) Computational tools for reconstructing neuronal morphology accurately and efficiently. Due to high densities of axonal and dendritic arbors of mammalian neurons in local brain regions, multiple labeled cells with dense neurites may appear fused in optical microscopy images, while existing automated tracing algorithms are incapable of resolving such junctions reliably and typically trace multiple cells as interconnected neuron clusters. Although a great deal of progress has been made in the other two aspects, there have been few efforts toward developing computational approaches that are required to more efficiently and accurately segment and characterize neuronal morphologies of many neurons in large brain volumes.

G-Cut leverages biological statistics and graph theory to automatically segment individual neurons with detailed neuronal morphologies from densely intermingled neuron clusters. It is rapidly and accurately performant in various CLARITY processed tissue blocks and Golgi-cox stained brain sections. Able to use a standard SWC file as input^23^, G-Cut can be easily applied on top of reconstruction results obtained from automatic, semi-automatic and manual tracing methods of different labs, making it a flexible and powerful component of an informatics pipeline. We combine G-Cut with automatic single neuron reconstruction methods (by APP2 in Vaa3D^24^, GTree^25^, NeuronStudio etc.) for a proposed workflow (Fig. 1) that includes data acquisition, neuron cluster reconstruction and single neuron segmentation (see Fig. 1e, Supplementary Figs. 5 and 9 for more real image stack cluster reconstruction and neuron segmentation examples; also see Supplementary Movie). The proposed workflow would address the challenge of reconstructing populations of neurons from tissue blocks, improving cell type identification, neuronal circuitry mapping and single cell resolution brain atlas development.

Our proposed workflow with G-Cut can reconstruct individual neurons from neuron clusters labeled with a wide range of techniques (rabies virus, genetic expression, etc.) that are much less challenging than “sparse” labeling strategies. In addition, as shown in this report, the G-Cut workflow can reconstruct Golgi-labeled individual neurons (Golgi labeling is notoriously for its noisy background and numerous neurites). This is significant because currently Golgi-cox method is the most practical and robust method to reveal neuronal morphologies of mammalian species such as rats, monkeys, and humans, where technical and ethical barriers prevent the application of genetic or viral labeling. Furthermore, we designed G-Cut such that it can be used both individually and integrated into large-scale informatics pipelines; therefore, it can be used systematically and comprehensively to reconstruct neurons and establish databases for comparison with neuronal cell types collected in NeuroMorpho.org. Overall, G-Cut is a robust software for practical and broad use in reconstructing neuronal morphology with a high potential to accelerate and scale-up processes of cell type classification, multi-compartmental modeling, and brain mapping.

A final note is that we have not tested the capability of G-Cut in reconstructing long axons in whole brain volumes. Axons display very distinctive morphological features from dendritic arbors in their complexity, such as length and thickness, numbers of dendritic branches and axonal collateral trajectory, as well as fine detailed morphological specificities (i.e., dendritic spines versus axonal varicosities, boutons of passages, or terminal boutons). Therefore, to accurately reconstruct axons with this detailed information (not just as curve skeletons) requires different mathematic strategies. Further, accurate reconstruction of individual axonal morphologies in whole brain context rely heavily on imaging technologies that achieve a good balance of sufficient resolution, reasonably good throughput, and relatively “sparse labeling” of individual axons in order to produce meaningful results. Considering the above challenges, accurate and simultaneous reconstruction of long projecting axons from many neurons in the whole brain context is a task better tackled by combined efforts from reconstruction pipelines and image acquisition techniques in future research.

## Methods

In G-Cut, we assume that a digitally reconstructed neuron cluster has been extracted from raster images and the neuronal cell body locations are known. Reconstructed neuron clusters can be obtained through one of the many currently available neuron tracing algorithms. Traced clusters are conventionally represented by a connected graph, from which individual neuron segmentation is achieved by an optimal graph partition that yields maximum global fitness in the graph. Currently, the input formats supported by G-Cut include (1) SWC file format (the current standard storage format for neuronal morphologies) and (2) vertex and edge list. From an initially reconstructed neuron cluster, represented as a graph (described by SWC file format or the more general vertex and edge list format), we define four types of nodes connected by edges: soma nodes, branch nodes, leaf nodes and path nodes. Soma nodes represent somas. Branch nodes are nodes with more than two immediate neighbors. Leaf nodes are nodes with exactly one immediate neighbor. These three types of nodes are of topological importance and are therefore called topological nodes. Path nodes are nodes with exactly two immediate neighbors. Associated with three-dimensional coordinates and connected by edges, topological nodes and path nodes constitute the geometric components of the connected graph representing a traced neuron cluster. We formally define a branch as a curve immediately connecting two topological nodes in the connected graph (Fig. 2a). It is important to note that, to the best of our knowledge, with the exception of the TREES Toolbox and NeuroGPS-TREE, all automatic tracing algorithms assume the presence of no more than one soma in a traced volume. Therefore, all traced branches correctly belong to the single soma. The SWC format echoes this single soma assumption by representing the traced structure as a tree graph, where all edges (branches) orient away from one root node (assumed single soma). By contrast, G-Cut considers image volumes where the single soma assumption does not hold. A tree graph representation of such a multi-soma neuron cluster contains branches with incorrect orientation: a branch’s orientation designated by the tree graph is false, whenever the branch does not actually belong to the assumed single soma, and therefore orients away from an incorrect root. It is easy to see the edge directionality intrinsic to tree graphs is no longer a meaningful representation of the biological data in a multi-neuron cluster. Therefore G-Cut discards such direction information altogether, and instead views the input as an undirected graph with numerous possible combinations of branch to soma assignments: a system with *n* branches and *m* somas gives rise to *n*^*m*^ total combinations, though we are able to greatly reduce this value by applying biologically relevant constraints. From all those possible combinations, G-cut identifies a single combination of branch soma assignments that maximizes the global fitness of the graph. Because each branch uniquely belongs to one and only one soma, at the completion of the assignment, segmentation of single neurons from a neuron cluster is achieved (Fig. 2d).

Our algorithm in G-Cut has three major steps:

To begin with, we define a morphological feature that we refer to as the Growth Orientation Feature (GOF) and derive the statistical distribution of the GOF using the existing experimental reconstruction of neuronal morphologies. We further compute fitness, as well as penalty, the complement of fitness, for any given branch-soma pair in the graph by incorporating GOF and branch length.

Secondly, we simplify the problem by eliminating branches whose soma assignment can be determined from the topology of the graph alone. Briefly, when traversing the graph from a given soma node, if a leaf node is encountered, all branches between the soma node and the leaf node can only originate from the given soma. They are subsequently assigned to the soma and excluded from further consideration. We next run Dijkstra’s algorithm for each pair of soma nodes in the graph, with one node being source and the other being target. The edge cost to leave a non-source soma node is set to infinity. The branches along the identified path, designated as common path, are reachable from either soma of the pair. Their soma assignments are therefore ambiguous. A given soma, with its neighbor somas reachable from it via a common path, and the minimum set of common paths connecting these neighboring somas, is considered as an independent unit of the neuron cluster graph. Branch assignments within the unit have no effect on the branch assignments outside of the unit. Each independent unit is then considered by the next stage of G-Cut.

In the final step, we derive one directed acyclic graph (DAG) from each soma in the independent unit, representing the maximum probable set of branches originating from the soma within the unit. In every such DAG, the GOF based penalty is computed and assigned as edge cost. We mathematically express topological relationships between branches in the unit DAGs as several linear equality and inequality constraints. With linear programming, we minimize the total penalty of all branch-soma pairs. The branch-soma assignments at the global minimum are then the solution to our segmentation problem.

We now describe each of the 3 stages in greater detail.

### Mathematic model for neuronal morphology

In a graph representation of a neuron cluster, branches reachable from multiple somas cannot be assigned to a unique soma based on topological relations alone. To overcome this difficulty and to approximate the empirical observation that branch orientation crucially guides successful manual tracing, we define and derive GOF as a data driven optimality estimator in the graph.

The GOF describes the orientation of a branch with respect to a given soma (Fig. 2b). A branch can be abstractly represented as a parametric curve in the three-dimensional space **C**(*l*): *R*_*l*≥0_ → *R*^3^ where *l* is the trajectory length from a starting point to a curve point and *R*^3^ is the three-dimensional coordinates of the point. For a branch of total length *L*, **a** = **C**(0) is the start and **b** = **C**(*L*) is the stop (Table 1).

**Table 1:** Mathematical symbols and their corresponding meanings

Symbols	Meaning
**C**	Neurite curve
**s**	Soma node
_*pi*_	Path node *і*
**v**_*i*_	Direction vector from a parent of path node *і* to the node itself
**a**, **b**	Two end nodes on a neurite curve
*g*	Penalty score
*w*	Degree of membership

We define the GOF of a branch **C** with respect to a soma **s** as below:

1 $${\mathrm{GOF}}\left( {{\bf{C}},{\bf{s}}} \right) = \frac{1}{L}{\int_{0}^{L}} {\theta \left( l \right)dl}$$

2 $$\theta \left( l \right) = {\bf{arccos}}\left\langle {\frac{{{\bf{C}}\left( l \right) - {\bf{s}}}}{{\left| {{\bf{C}}\left( l \right) - {\bf{s}}} \right|}},{\bf{C}}\prime \left( l \right)} \right\rangle$$

where *θ*(*l*) is the angle between the tangent vector **C**′(*l*) of the branch curve at **C**(*l*) and the unit vector (**C**(*l*) − **s**) × |**C**(*l*) − **s**|^−1 pointing from **s** to **C**(*l*). 〈·,·〉 denotes dot product of two vectors. Because **C**(*l*) is parametrized with curve length, **C**′(*l*) is always a unit vector. Please note that the direction of a branch depends on its topological relation with a given soma. GOF(**C**_**a**→**b**_, **s**) and GOF(**C**_**b**→**a**_, **s**) have distinct values.

Substituting Eq. (2) into Eq. (1), we calculate the GOF of a reconstructed branch as follows:

3 $${\mathrm{GOF}}\left( {{\bf{C}},{\bf{s}}} \right) = \frac{{\mathop {\sum }\nolimits_{j = 1}^n |{\bf{v}}_j|{\bf{arccos}}\left\langle {\frac{{{\bf{p}}_j^ \ast - {\bf{s}}}}{{\left| {{\bf{p}}_j^ \ast - {\bf{s}}} \right|}},\frac{{{\bf{v}}_j}}{{|{\bf{v}}_j|}}} \right\rangle }}{{\mathop {\sum }\nolimits_{j = 1}^n |{\bf{v}}_j|}}$$

4 $${\bf{p}}_j^ \ast = \frac{{{\bf{p}}_{j - 1} + {\bf{p}}_j}}{2}$$

5 $${\bf{v}}_j = {\bf{p}}_j - {\bf{p}}_{j - 1}$$

For a branch-soma pair with GOF = *x*, we use the tail distribution of GOF, TailDist(*x*) as a measure of the likelihood that the pair occurs in a biological system (Fig. 2c):

6 $${\mathrm{TailDist}}\left( x \right) = {\int_{x}^{\pi}} {{\mathrm{PDF}}\left( \rho \right)d\rho = 1 - {\mathrm{CDF}}\left( x \right)}$$

where function PDF is the probability density function of the GOF empirically constructed from the NeuroMorpho. Org database.

We also define fitness of a branch as below:

7 $${\mathrm{fitness}}\,\left( {{\bf{a}} \to {\bf{b}},{\bf{s}}} \right) = {\mathrm{weight}}\,\left( {{\bf{a}} \to {\bf{b}}} \right) \times {\mathrm{TailDist}}\left[ {{\mathrm{GOF}}\left( {{\bf{a}} \to {\bf{b}},{\bf{s}}} \right)} \right]$$

where **a** → **b** denotes a branch that starts at **a** and ends at **b**. weight(**a** → **b**) is the weight parameter of a branch **a** → **b** determined by its length. This term is used to suppress noise from very short branches (a common tracing artifact from noisy image stacks) on the graph.

Our segmentation method relies on Dijkstra’s algorithm and linear programming. Both algorithms search for a global minimum. Therefore, we convert the fitness score fitness(**a** → **b**, **s**) to penalty score *g*_**a**→**b**,**s**_:

8 $$g_{{\bf{a}} \to {\bf{b}},{\bf{s}}} = {\mathrm{weight}}\left( {{\bf{a}} \to {\bf{b}}} \right) \times \left\{ {1 - {\mathrm{TailDist}}\left[ {{\mathrm{GOF}}\left( {{\bf{a}} \to {\bf{b}},{\bf{s}}} \right)} \right]} \right\}$$

### Transforming neuron clusters into a directed acyclic graph

In a reconstructed neuron cluster, some branches join multiple somas while others do not. The segmentation problem can be simplified by focusing on branches connecting several somas. This can be done with breadth first search (BFS) starting at leaf nodes and terminating at soma nodes. If only one soma node can be reached from the leaf node, all branches between the soma node and the leaf node can only originate from the encountered soma. They are subsequently assigned to the soma and excluded from further consideration.

The GOF tells us how likely a branch has grown from a soma based on the branch’s orientation and its relative position with respect to a soma. However, other factors must also be considered. First, the GOF depends on the direction of a branch. Edges in our input graph are undirected. A branch with end nodes **a** and **b** can be considered either from **a** to **b** or from **b** to **a**. Which direction should it be? Second, branches are in a graph and they have grown from the soma, so their direction must be constructible with a growth process. Here we present how to determine potential branch directions for a certain soma from an undirected network. Our process is based on the following rules. First, if a branch has grown from a soma, then there must be a directed path from the soma to the branch and all branches on the path must have grown along the same direction of the path. Second, if there are multiple directed paths to a branch, we choose the most likely one. We consider the directed path with the lowest sum of penalty as the most likely one. Based on these two rules, we employ Dijkstra’s algorithm^26^ to determine branch direction and path from soma (Supplementary Note 3).

Please note that the Dijkstra’s algorithm needs a soma to be assigned as the root. We run it independently for each soma in the graph. Given a root soma, it not only outputs the minimal total cost from the soma to reach each branch, but also, more importantly, the directed path to the branch by recording the previous branches for each branch. In this way, the graph which represents the neuron cluster is converted to multiple trees rooted at their respective somas, and the penalty along the path direction can be assigned as weight to each branch in the tree (Fig. 2d).

### Segmentation with linear programming

We formulate the graph partition problem as a linear programming problem^27^.

Dijkstra’s algorithm constructs an independent, directed path to branches from each soma. To determine from which soma a branch originates, we evaluate all possible branch soma pairs. We quantify the degree of membership for a branch **C** belonging to a soma **s** as *w*_**C**,**s**_. Assigning branch **C** to soma **s** is often not an isolated operation: this assignment dictates that all branches occurring earlier along the path from **s** to **C** are also assigned to **s**. We therefore consider *w*_**C**,**s**_ for all branch soma pairs jointly to minimize a global penalty function for the whole graph:

$$E = \begin{array}{*{20}{c}} {{\it{arg}}{\mathrm{min}}} \\ {{\mathbf{s}} \in {\mathbf{S}}} \end{array}\sum \langle {\mathbf{w}}_{ \cdot ,{\mathbf{s}}},{\mathbf{g}}_{ \cdot ,{\mathbf{s}}}\rangle \,{\mathrm{where}}$$

$${\bf{w}}_{ \cdot ,{\bf{s}}}\,{\mathrm{is}}\,{\mathrm{a}}\,{\mathrm{column}}\,{\bf{s}}\,{\mathrm{of}}\,{\mathrm{matrix}}\,{\bf{W}} = \{ w_{{\bf{C}},{\bf{s}}}\}$$

$${\bf{g}}_{ \cdot ,{\bf{s}}}\,{\mathrm{is}}\,{\mathrm{a}}\,{\mathrm{column}}\,{\bf{s}}\,{\mathrm{of}}\,{\mathrm{matrix}}\,{\bf{G}} = \{ g_{{\bf{C}},{\bf{s}}}\}$$

Under the following constraints:

9 $$\mathop {\sum}\nolimits_{{\bf{s}} \in {\bf{S}}} {w_{{\bf{C}},{\bf{s}}} = 1}$$

10 $$w_{{\bf{C}},{\bf{s}}} \ge 0$$

11 $$w_{{\bf{C}},{\bf{s}}} \le w_{{\mathrm{par}}({\bf{C}},{\bf{s}}),{\bf{s}}}$$

In the expression, *E* is the energy function representing total penalty of all soma-branch pairs considered by the algorithm. **S** is a set representing all somas. **G** is a penalty matrix where *g*_**C**,**s**_ is the penalty of a soma-branch pair **C** and **s**. **W** is a membership value matrix of each branch to each soma. par(**C**, **s**) represents the parent branch of a branch **C**.

The constraints expressed by Eq. (9) and Eq. (10) guarantee that the membership of a branch to a soma is non-negative and its total membership to all somas is 1.

Importantly, the constraint in Eq. (11) reflects the tree topology of a neuron. Because a downstream branch grows from an upstream branch, its membership to the root soma **s** cannot be higher than its immediate upstream branch. In this way, we enforce the correct topology structure into our optimization problem.

After we use linear programming to achieve optimization on the graph partition problem, we assign the branches to a certain soma according to assignment rule:

12 $${\mathbf{s}}^ \ast = \begin{array}{*{20}{c}} {{\it{arg}}{\mathrm{max}}} \\ {{\mathbf{s}} \in {\mathbf{S}}} \end{array}{\mathbf{w}}_{{\mathbf{C}},{\mathbf{s}}}$$

The segmentation is completed when all branches have been assigned to specific soma.

### Tissue preparation

All experiments in this project were conducted according to the regulatory standards set by the National Institutes of Health Guide for the Care and Use of Laboratory Animals and by the institutional guidelines set by the Institutional Animal Care and Use Committee at USC or University of California in Los Angeles (UCLA). Brain tissues in this study are from adult mice (2 month-old male C57BL/6J) that either received injections of G-deleted rabies-eGFP virus (Salk vector core) or with genetic-based sparse labeling using the MORF method^22^. Mice were pair-housed within a room that was controlled for temperature (21–22 °C), humidity (51%), and light (12 h light:12 h dark cycle with lights on at 6:00 a.m. and off at 6:00p.m.). Subjects had ad libitum access to tap water and mouse chow throughout the experiments. Rabies injection surgeries were performed in a BSL-2 level environment and performed by individuals who had been rabies-vaccinated. Following surgery, rabies-infected animals were individually housed in a separate BSL-2 level facility for 4–7 days. Then each animal was deeply anesthetized with an overdose injection of sodium pentobarbital and trans-cardially perfused with approximately 50 ml of 0.9% saline solution followed by 50 ml of 4% paraformaldehyde (PFA; pH 9.5). The brains were removed and post-fixed in 4% PFA for 24–48 h at 4 °C.

These brains were then processed with CLARITY^28^. In brief, these brains were sliced into 1 mm thick coronal sections and immersed in SDS solution at 37 degrees Celsius for three weeks or until clear. Tissue sections were then placed into graded imaging solutions of 2’2- thiodiethanol^29^ with the final 64% TDE imaging solution matching the refractive index of CLARITY tissue.

CLARITY tissue sections were imaged using an Olympus FVMPE-RS multiphoton microscope which uses both Mai Tai and Insight laser excitation. For Z-stack imaging of rabies-eGFP or genetically labeled neurons, Mai Tai laser excitation was set to 920 nm to visualize rabies eGFP signal. For the 500 μm Z-stack shown in Fig. 1a, the infralimbic cortex (ILA) was imaged at 1024 × 1024 resolution using a 25× Olympus objective (XLPLN25XWMP2) at 10 μs/pixel with a 1 μm Z-slice. The resulting 3D image volume has an XYZ pixel resolution of 0.497 × 0.497 × 1μm.

Golgi-cox staining was performed following a protocol modified from previous reports^30,31^. Mouse brains were sliced into 150 µm thickness for Golgi-cox impregnation. Images for 3D rendering were collected using Olympus VS120 Virtual Microscope under ×30 silicon oil objective lens at 1 µm z steps.

### Reporting summary

Further information on experimental design is available in the Nature Research Reporting Summary linked to this article.

## Supplementary information

Supplementary Information

Description of Additional Supplementary Files

Supplementary Movie 1

Supplementary Software 1

Reporting Summary

Source Data

## Data Availability

The raw image data generated for the study (shown in Figs. 1, 6, 7 and Supplementary Fig. 9) are available from corresponding authors on request. The source data underlying Figs. 3, 5a–c, 6b,g and 7g and Supplementary Figs. 1, 3, 4, 5e and 9 are provided as a Source Data file.

## Code availability

G-Cut is open source and available as Supplementary Software written in Matlab code. All source code is freely available for noncommercial use (https://muyezhu@bitbucket.org/muyezhu/gcut.git).
