# Supplementary material for: Precise segmentation of densely interweaving neuron clusters using G-Cut
Source: Nat Commun. 2019 Apr 4;10:1549. doi: 10.1038/s41467-019-09515-0 (PMC6449501; doi:10.1038/s41467-019-09515-0)
Supplement: Supplementary file 1 — Supplementary Information [file 41467_2019_9515_MOESM1_ESM.pdf]

Supplementary Information

Precise Segmentation of Densely Interweaving neuron clusters using G-Cut

Li *et al.*

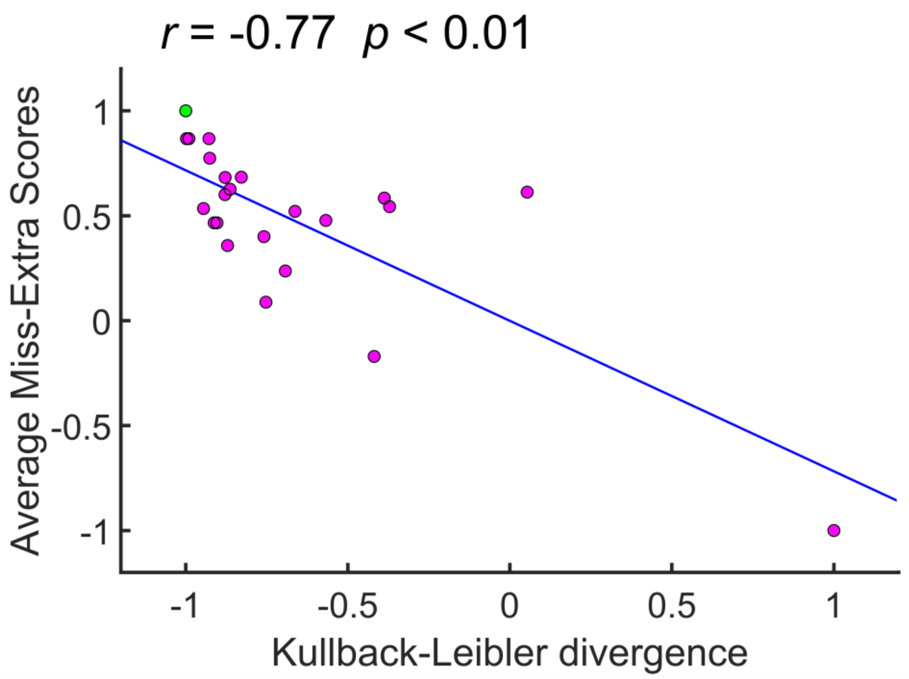

Supplementary Figure 1. Average MES of G-Cut by using different GOF distributions on a Golgi-staining image stack. The image stack was obtained from mouse neocortex and the neuron clusters in it were reconstructed by Neuronstudio as shown in Fig. 7c. Manual reconstruction shown in Fig. 7b was used as ground truth. The green dot indicates the highest average MES of G-Cut, achieved by using GOF distribution derived from data set of mouse neocortex. Magenta dots indicate the average MES of G-Cut by using GOF distribution derived from other data sets, such as other mouse brain regions (basal ganglia, brainstem, cerebellum, hippocampus, hypothalamus, pons, retina, spinal cord, thalamus, and ventral striatum) and different species (*C elegans*, drosophila melanogaster, human, monkey, mouse, rat, spiny lobster, and zebrafish) The horizontal axis shows the Kullback-Leibler divergence between GOF distribution of mouse neocortex (green dot) and others (magenta dot). Pearson correlation test shows the correlation between average MES and KL-divergence is significant. Results were standardized for visualization. Source data are provided as a Source Data file.

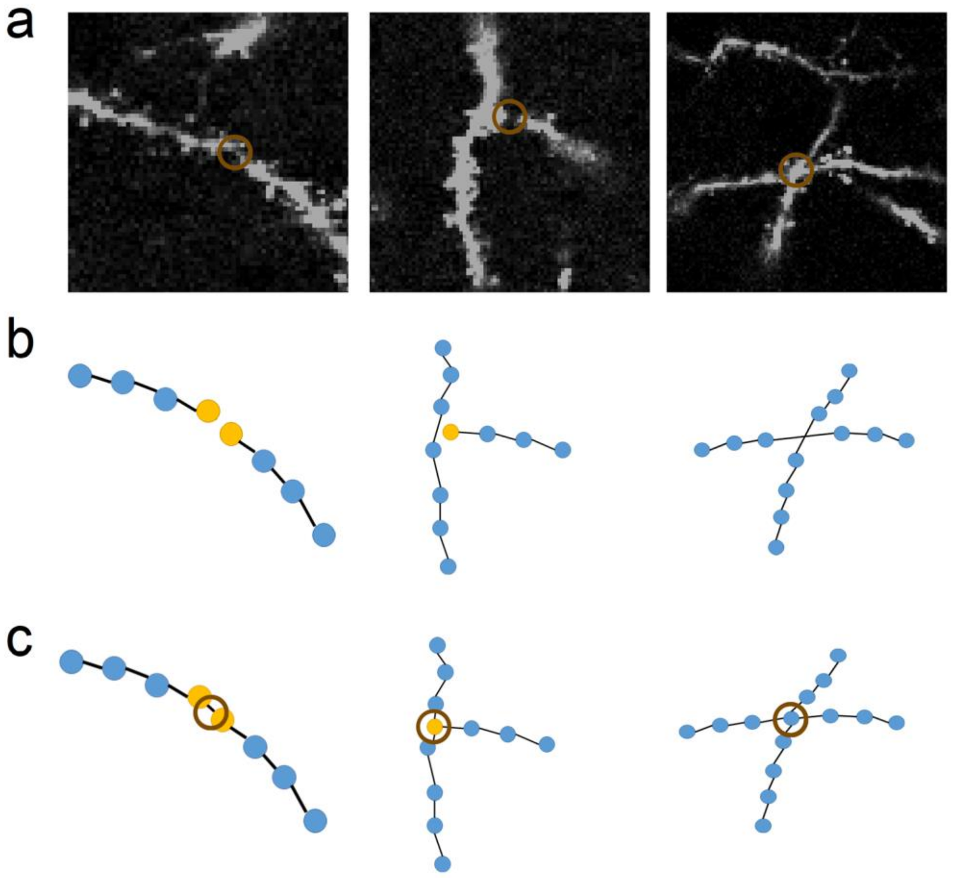

Supplementary Figure 2. Spurious links in a real image stack and their topological connection changes in digital reconstruction. The spurious links between two different branches appear in three situations: first, in the end of each branch; second, in the end of one branch and in the segment of another branch; third, in the segment of each branch. **a** shows spurious links in the three situations in a real image stack. The branches are shown in white pixels on black background and spurious links are drawn in brown circles. In **b**, the correct topological connections correspond to the three situations are shown. **c** shows the topological changes between branches in the three situations.

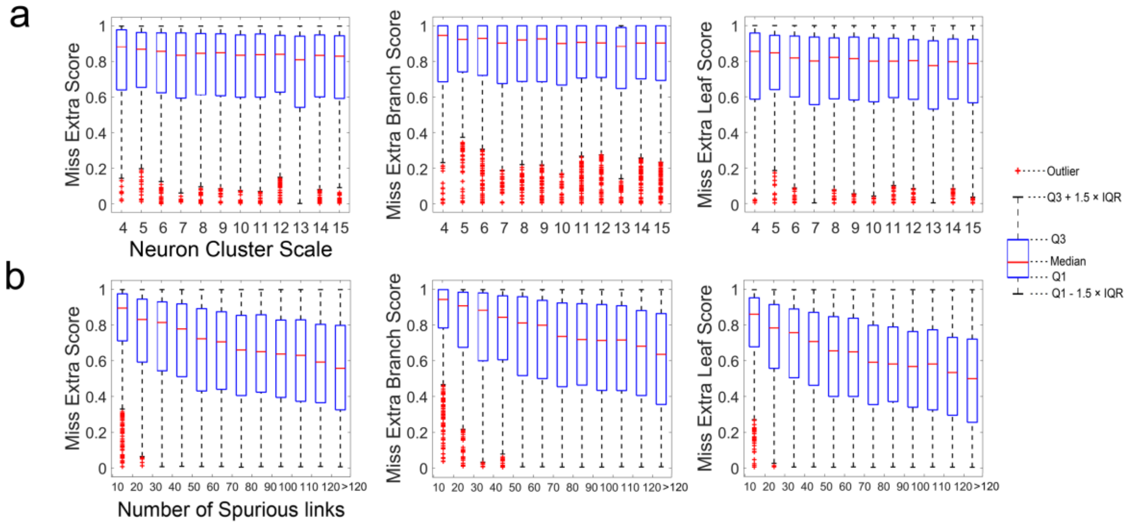

Supplementary Figure 3. a Boxplot of Miss-Extra-Scores between G-Cut segmented and ground truth neurons across different neuron cluster scales. The cluster scale ranges from 4 to 15. The red line represents median MES. **b** Boxplot of G-Cut MES in cluster scale six with different degrees of entanglement. Number of total spurious links in a cluster lies in intervals shown along the x-axis. Source data are provided as a Source Data file.

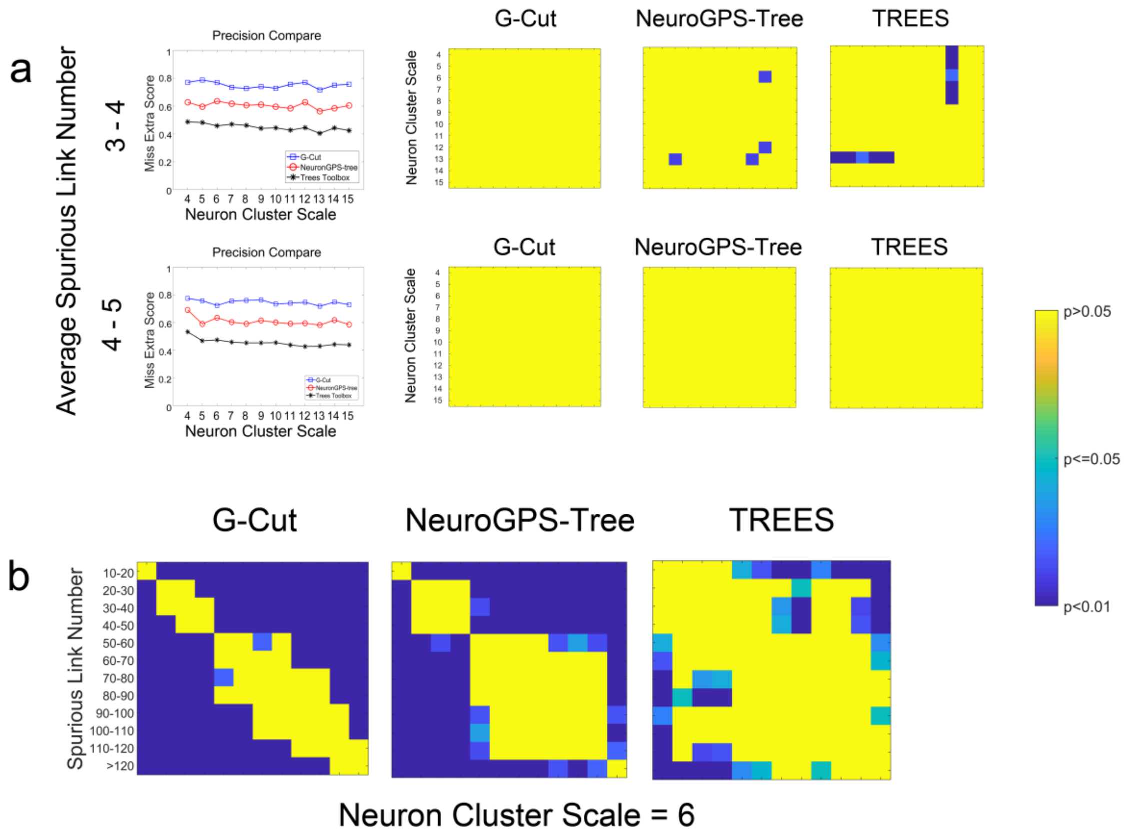

Supplementary Figure 4. Statistical analysis of MES results from G-Cut, NeuroGPS-Tree and TREES toolbox in simulated datasets. **a** Neuron cluster scale does not strongly influence segmentation accuracy. In order to avoid interaction between cluster scale and degree of entanglement in statistical tests, we first group clusters according to their average number of spurious links per neuron into intervals of 1. In the resulting cluster groups, we further examine clusters with per neuron spurious links of [3, 4) and [4, 5) (other cluster groups occur at much lower frequency and may not satisfy sufficient sample size for all scales). Top left graph show the average MES of clusters with per neuron spurious links in [3, 4) from the three methods (the total number of neuron clusters is 3753). Upon visual inspection, there is no obvious correlation between MES cluster scale. Kruskal-Wallis test does show significant difference between MES of G-Cut segmentation at different scales ($H(11) = 25.801$, $p = 0.007$). However, pair-wise Mann-Whitney U-test with Benjamini–Hochberg correction show no significant difference between MES of any two cluster scales following G-Cut segmentation, and few significant pairs following

NeuroGPS-Tree and TREES toolbox segmentation (top right panel). Similarly, we see no obvious correlation between MES and cluster scale when per neuron spurious links fall in [4, 5) (bottom left graph, the total number of neuron clusters is 5093). Kruskal-Wallis test has a non-significant p-value of 0.064 for G-Cut segmentation ($H(11) = 18.819$), while Mann-Whitney U-test with Benjamini–Hochberg correction shows no cluster scale pair to be significantly different in MES following segmentation by any of the three methods (bottom right panel). These results suggest that when cluster degree of entanglement is tightly controlled, cluster scale does not strongly influence segmentation accuracy. **b** The p-values of pair-wise Mann-Whitney U-test MES results with Benjamini–Hochberg correction derived from neuron clusters of different degrees of entanglement. The axis shows the range of spurious link number when the cluster scale is six. Kruskal-Wallis test shows significant difference between MES of G-Cut segmentation at different degree of entanglement ($H(11) = 543.291$, $p < 0.01$). And MES results of NeuroGPS-Tree and TREES toolbox are also significantly different at different degree of entanglement (Kruskal-Wallis test, $H(11) = 307.698$, $p < 0.01$, and $H(11) = 51.533$, $p < 0.01$ for NeuroGPS-Tree and TREES toolbox, respectively). Source data are provided as a Source Data file.

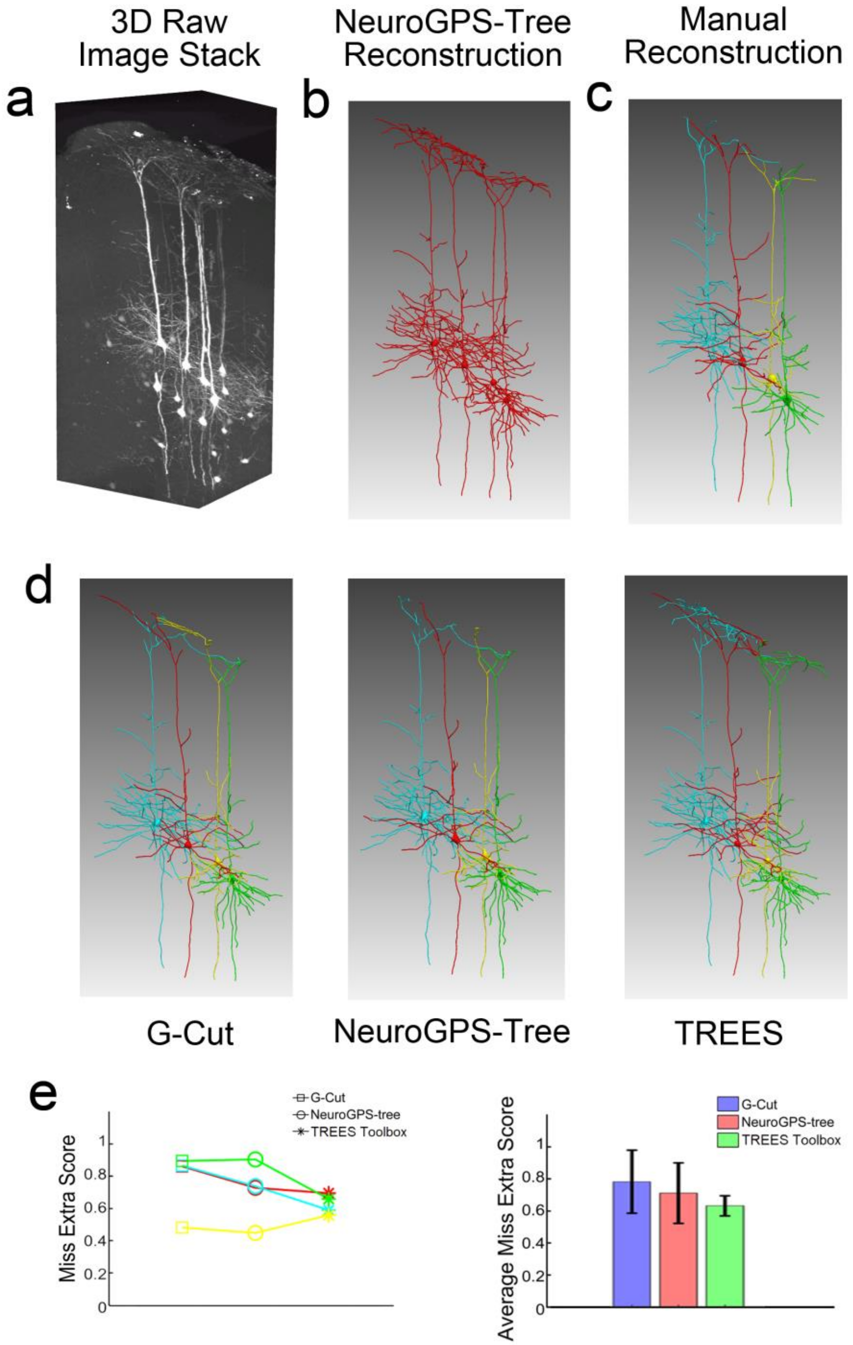

Supplementary Figure 5. Comparison of reconstruction results by G-Cut, NeuroGPS-Tree and TREES. **a** The raw image stack (test data used by NeuroGPS-Tree software). Data size 896×348×200 voxels. **b** A neuron cluster was reconstructed from image stack using GTree software, a latest release based on NeuroGPS-Tree. **c** Four neurons with distinguishable dendritic trees were manually reconstructed from image stack with neuTube software and used as ground

truth. **d** The neuron cluster was segmented by G-Cut, NeuroGPS-Tree and TREES toolbox into four individual neurons, respectively. Identical post-processing (see Supplementary Figure 7 and Supplementary Note 2) was applied on segmentation results from all three algorithms. **e** Miss-Extra-Scores of the segmented neurons from the three methods. Different neurons are represented by different colors. MES of neurons segmented by G-Cut, NeuroGPS-tree and TREES toolbox is represented by square, circle, and asterisk, respectively. Source data are provided as a Source Data file.

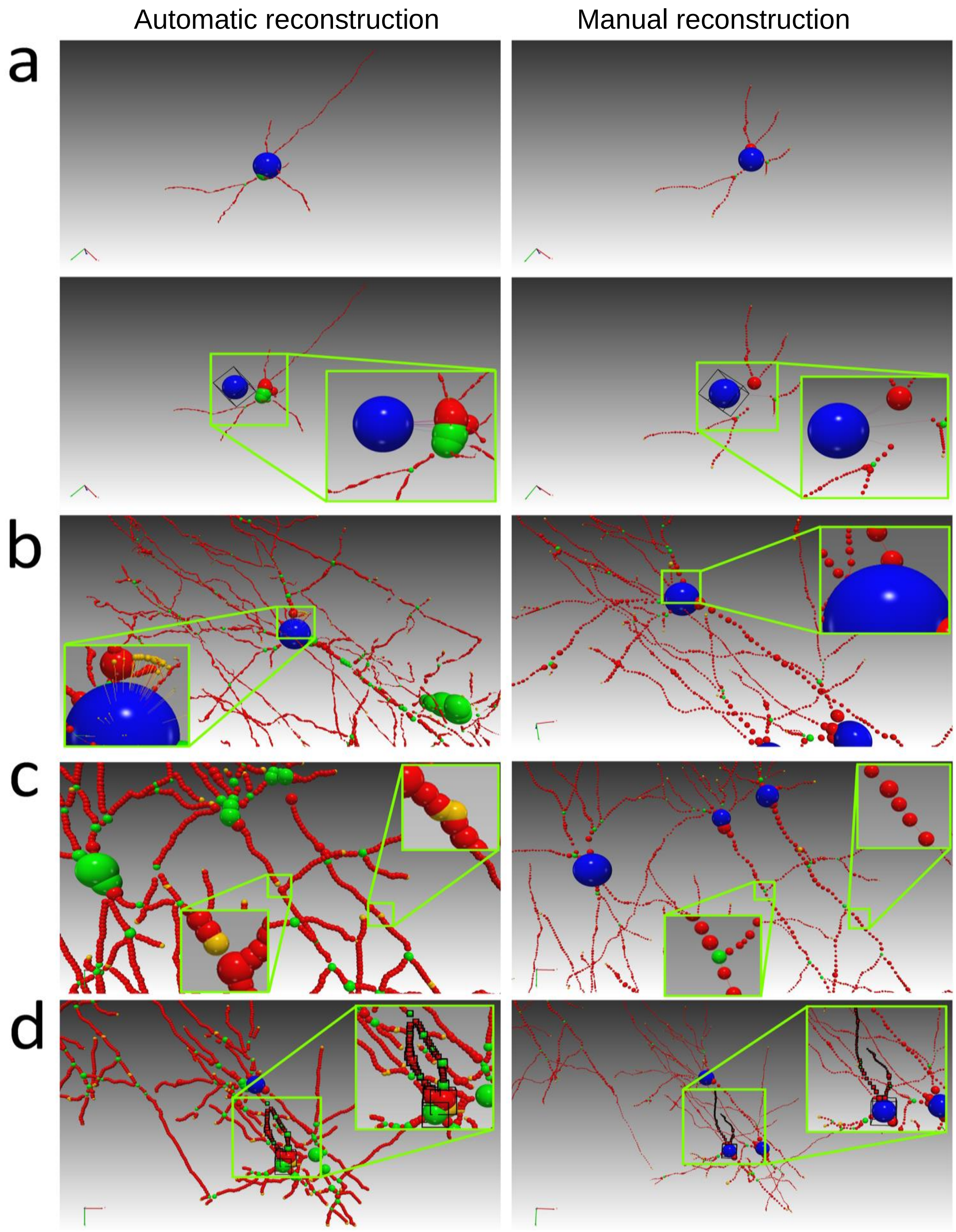

Supplementary Figure 6. Example of four different tracing errors. The four tracing errors result in different topological connections between automatic reconstruction neurons and manual reconstruction neurons. In **a**, the automatic reconstruction neuron (upper left) is visually similar to manual reconstruction neuron (upper right). But the automatic reconstruction neuron has a

tracing error in soma indicated by the yellow-green box (bottom left) compared with manual reconstruction. In **b**, the automatic reconstruction neuron has several short and thin branches connected with soma (indicated by the yellow-green box). But manual reconstruction neuron is smooth. In **c**, yellow-green box show some breaks in branches of automatic reconstruction neurons which are connected in manual reconstruction neurons. These breaks result in different topological connections between neurons. **d** shows a tracing error by automatic reconstruction method in a soma (indicated by the yellow-green box and black boxes). The soma is divided into several nodes which is only one soma node in manual reconstruction and these nodes are connected by a branch path indicated by the black boxes. The branch path should be two different branches coming out from the soma in manual reconstruction. Thus in this situation we cannot simply merge the branch path and soma nodes into one soma.

a

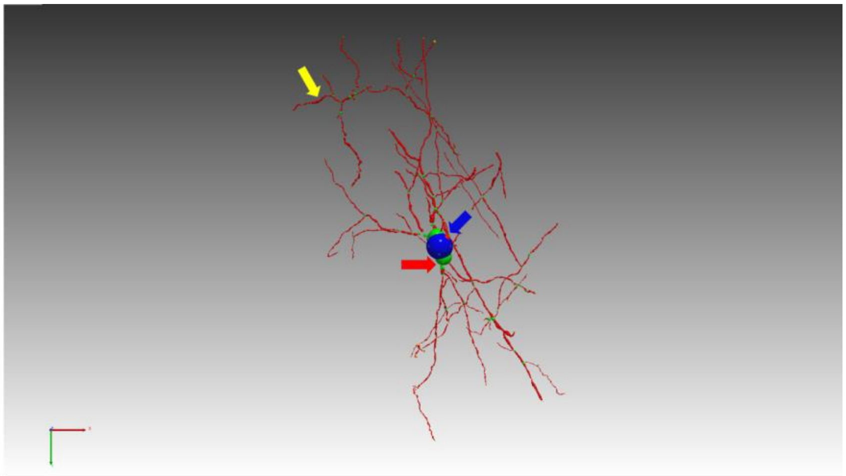

b

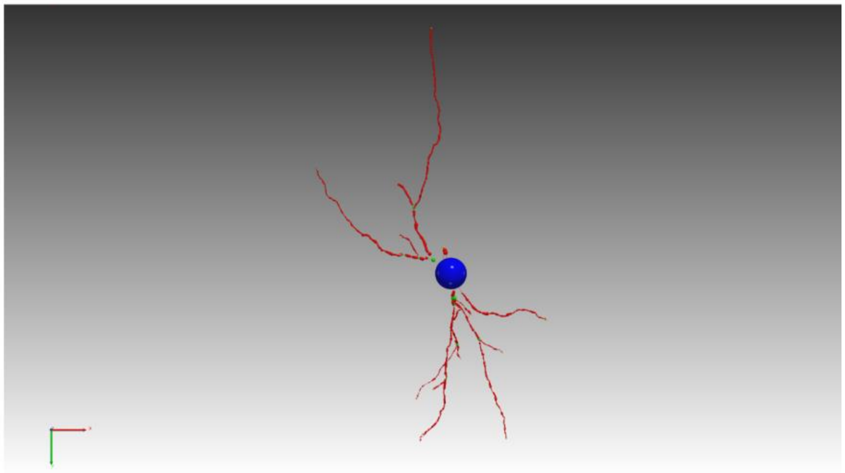

Supplementary Figure 7. Two tracing errors (indicated by red and blue arrows) and redundant branches (indicated by a yellow arrow) are shown in **a**. The tracing error shown by the blue arrow is the same as Supplementary Figure 6b and the tracing error shown by the red arrow is the same with Supplementary Figure 6a. We developed two methods to solve the tracing errors. To solve tracing errors shown by the blue arrow, we will detect these short and thin branches near the soma according to their distance and average diameter, and then merge these branches with the soma into a new soma node. For tracing errors shown by red arrows, we will detect the nodes inside the soma node and check for two conditions: (1) whether they are directly connected with the soma or (2) the path between them and the soma are also inside the soma node. If the nodes meet one of the two conditions, they can be merged with the soma. Tracing errors in Supplementary Figure 6d will be considered in our further developments. For the redundant branches, we prune them using

methods described in Supplementary Figure 8 and Supplementary Note 2. **b** shows the result after tracing errors were fixed and redundant branches were pruned.

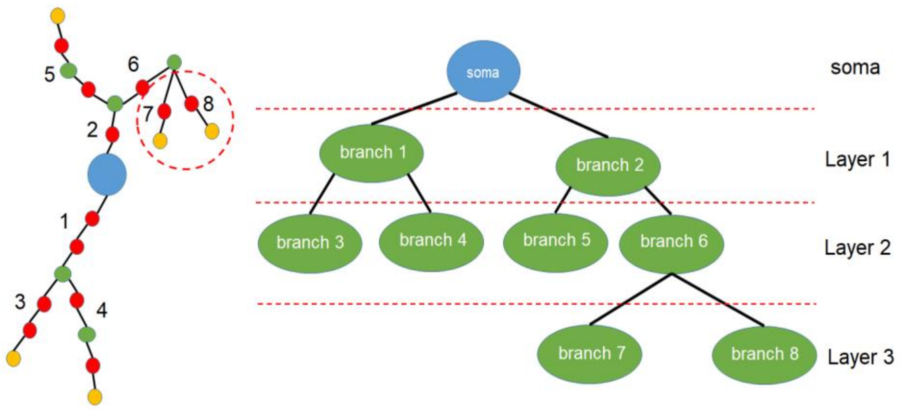

Supplementary Figure 8. Detecting and pruning redundant branches in a neuron. In the left panel we show the tree structure of a neuron that has eight branches and redundant branches that need to be pruned (shown in the red circle). In the right panel we show a tree graph of the neuron. The node in the graph represents a branch, and links between branches are represented by connections between nodes. The connection between nodes in two adjacent layers indicates that the node in the upper layer is the parent node of bottom layer. We calculate GOF of all branches and prune the redundant branches according to the method in Supplementary Note 2.

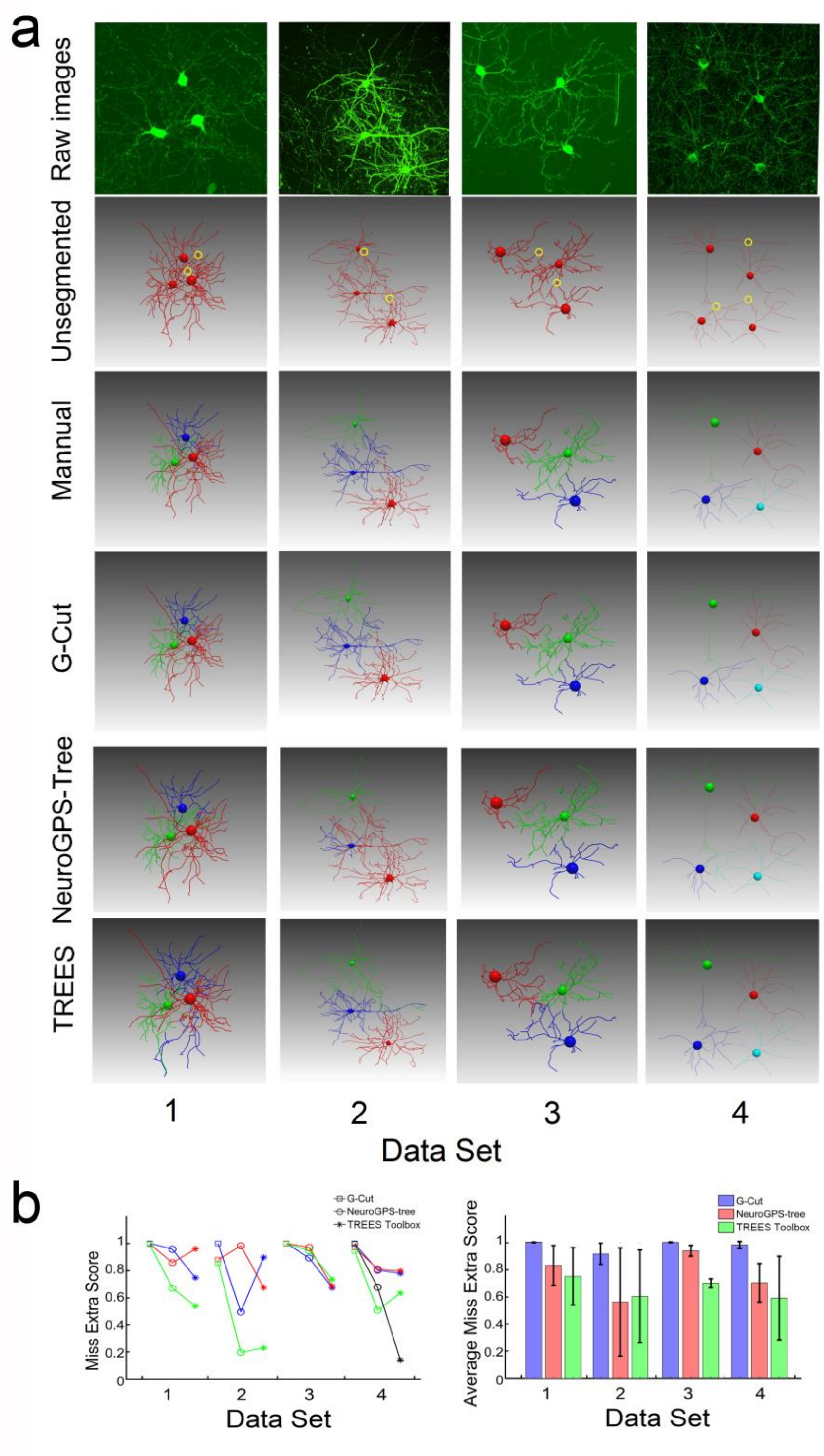

Supplementary Figure 9. Validation of G-Cut segmentation performance on four real image stacks. **a** Neuron clusters were reconstructed in Vaa3D software. Spurious links are drawn in yellow circles in the reconstructed neuron clusters. The reconstructed neuron clusters were

segmented by G-Cut, NeuroGPS-tree, TREES toolbox respectively, and compared to manually reconstructed ground truth. Different neurons in each data set are represented by different colors. **b** MES of neurons segmented by G-Cut, NeuroGPS-tree and TREES toolbox is represented by square, circle, and asterisk, respectively. The standard deviation is shown as error bar. Source data are provided as a Source Data file.

Supplementary Note 1. Simulation of neuron clusters

Due to the lack of publicly available reconstructed intact neuron clusters, we simulated neuron clusters by selecting and joining random subsets of 2693 well reconstructed neurons (including 435 interneurons and 2258 principal neurons) hosted on neuromorpho.org. In our synthetic data, the information of a neuron is represented by two parts: one part is a set of node information (including the node type, x, y, z location, and radius); and another part is an adjacency matrix representing connecting edges between nodes. We generated two datasets to evaluate the effect of cluster scale and degree of entanglement respectively. The procedures are listed as below:

1. <u>Starting neuron population</u>: Denote the predetermined number of neurons in a cluster as n, and the corresponding cluster scale as C_n. From the public dataset of well reconstructed neurons, we randomly chose m pyramidal neurons and n - m interneurons as starting neuron population, where $P(m = k \mid 1 \leq k \leq n) = (n - 1)^{-1}$. One of the n neurons is randomly selected as base neuron. Other neurons subsequently become connecting neurons. Each connecting neuron is joined with the base neuron as described in the following step 2 – 4. The process is iterated until all neurons are joined into a single cluster.
2. <u>Spurious link construction</u>: During the neuron tracing process, if the distance between branches of two neurons is very small, an automatic tracing method will erroneously connect the gap between the two branches into a spurious link. To realistically mimic real world applications of neuron cluster tracing, we placed cell bodies of connecting neurons at random locations in the same bounded volume space as the base neuron. If a pair of branches from different neurons have a distance to each other less than the sum of their radius, we considered the event an occurrence of spurious link and construct a connection between these two branches (as shown in Supplementary Figure 2).

3. Synthetic dataset with varying cluster scales: In order to understand how the cluster scale affects segmentation, it is necessary to bound the number of spurious links to a reasonable range. We first empirically derived a distribution for the number of spurious links between a random neuron pair, using criteria described in **2**. The neuron pair was randomly drawn from the set of well reconstructed neurons, and positioned together randomly 1000 time. We repeated the drawing and positioning 50 times, resulting in a total spurious link counts for 50,000 clusters. The result shows a majority of spurious links number is less than 10. The number of spurious link is extremely low when it is 1 and does not make sense in real image stacks. Thus, we bound the spurious number between a neuron pair to be between 2 and 10. We then iteratively join the $n - 1$ connecting neurons to the base neuron. For each connecting neuron, a random cell body position is generated and spurious link numbers are counted. If the number falls within 2 and 10, we accept the cell body position and construct links between the connecting and base neuron. Otherwise, a new position will be generated. The cluster formed from the joining operation will be considered as the new base neuron. The final cluster C_n then contains spurious links ranging between 2 * (n – 1) and 10 * (n – 1), to be assigned between n cell bodies. We generated 100 clusters for each cluster scale C_n. For clusters with the same scale, the difficulty of the segmentation problem will only differ up to a bounded constant factor, and no unusually dense entanglement can occur. This allows us to analyze how cluster scale affects segmentation accuracy.

4. Synthetic dataset with varying degrees of entanglement: In order to understand how cluster degree of entanglement affects segmentation accuracy, we used a fixed cluster scale, $n = 6$, for the entire dataset. Spurious link constructions were performed without an upper bound. We generated 10,000 clusters and stratified the cluster population based on probability distribution of spurious link number (Fig. 5c). From clusters with spurious link number in

each of the intervals [10, 20), [20, 30) … [120, ∞), we randomly drew 100 samples for analysis.

One example of the reconstructed neuron cluster is shown in Fig. 4.

Supplementary Note 2. The redundant branches pruning method.

We compute GOF of all branches in a neuron, and the total GOF of a branch *i* calculated by the equation:

$$\frac{\left(\sum \text{[illegible]} \left(\mathrm{GO} \text{[illegible]}\right) \text{[illegible]}\right)}{\sum \text{[illegible]}}$$

where *j* represents all child branches of a branch *i* and $length_i$ represents the length of a branch *i*.

After we calculate the total GOF of all branches of a neuron, we use a threshold value to prune the branches. The threshold can be a constant value or variable according to each neuron. If the total GOF of a branch is larger than the threshold value, all of its child branches are discarded from the neuron.

Example: As shown in Supplementary Figure 8, branch 7 and branch 8 are the child branches of branch 6 in the right figure. If the total GOF of branch 6 is larger than a threshold value, branch 7 and branch 8 are discarded from the neuron.

Supplementary Note 3. The Dijkstra's algorithm for branch orientation

Input: A geometric network V and a soma $\mathbf{s}$

Output: Total Cost, LocalCost, PreviousBranch assigned to each

Procedure:

For each branch $\mathbf{C}$ in V

Assign ∞ to TotalCost($\mathbf{C}$)

Assign ∞ to LocalCost($\mathbf{C}$)

Assign NULL to PreviousBranch($\mathbf{C}$)

End

Assign 0 to TotalCost($\mathbf{s}$)

Push s into a Heap

While the Heap is not empty

Pop out the node whose TotalCost is the smallest in the Heap. We call this node op.

If is $\mathbf{s}$ or it is not a soma node, then

For each branch which directly connects to with a node

Calculate penalty g on branch

If TotalCost(top)+g $_{\mathbf{b},\mathbf{s}}$ < TotalCost($\mathbf{C}_i$), then

Assign to LocalCost($\mathbf{C}_i$).

Assign TotalCost(top)+g to TotalCost($\mathbf{C}_i$).

Assign to PreviousNode($\mathbf{C}_i$).

End

If is not in the Heap, then

Push into the Heap.

End

End

End

End
