# Supplementary material for: Precise segmentation of densely interweaving neuron clusters using G-Cut
Source: Nat Commun. 2019 Apr 4;10:1549. doi: 10.1038/s41467-019-09515-0 (PMC6449501; doi:10.1038/s41467-019-09515-0)
Supplement: Supplementary file 2 — Description of Additional Supplementary Files [file 41467_2019_9515_MOESM2_ESM.pdf]

Description of Additional Supplementary Files

File Name: Supplementary Movie 1
Description: This movie shows an example of using G-Cut to simultaneously segment multiple neurons labeled with rabies viral tracers.

File Name: Supplementary Software 1
Description: G-Cut is open source and available as Supplementary Software written in Matlab code. All source code is freely available for noncommercial use (*https://muyezhu@bitbucket.org/muyezhu/gcut.git*).
